# Supplementary figures and images for: Genome-wide proximity between RNA polymerase and DNA topoisomerase I supports transcription in Streptococcus pneumoniae
Source: PLoS Genet. 2021 Apr 30;17(4):e1009542. doi: 10.1371/journal.pgen.1009542 (PMC8115823; doi:10.1371/journal.pgen.1009542)

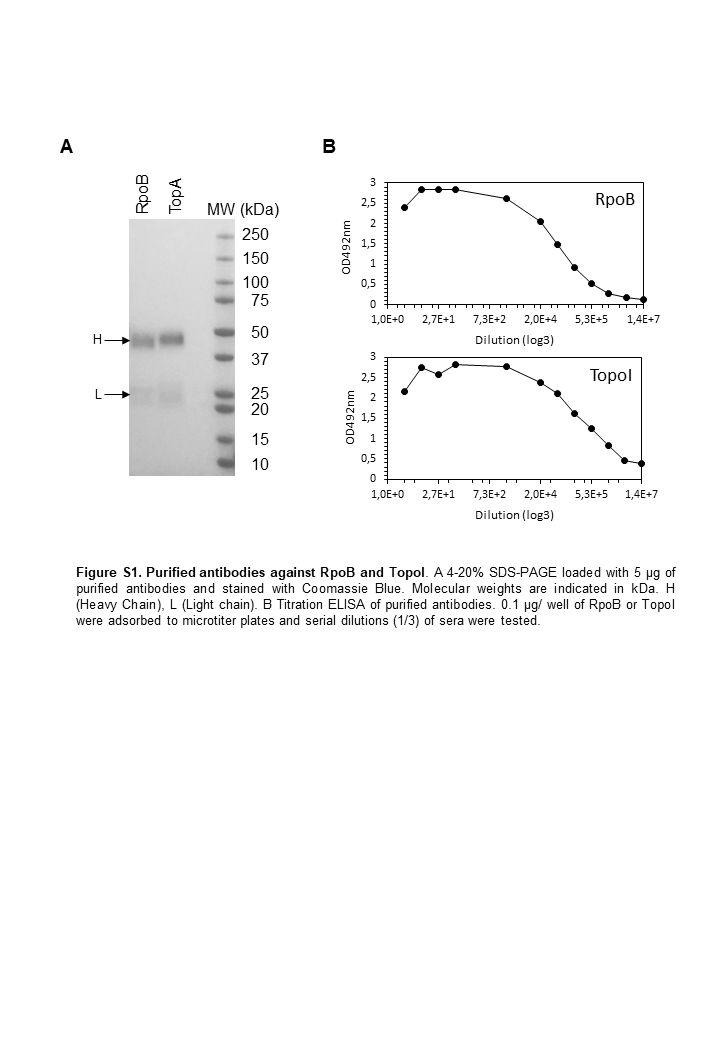

Supplement: S1 Fig — A) Purified antibodies against RpoB and TopoI. A 4–20% SDS-PAGE loaded with 5 μg of purified antibodies and stained with Coomassie Blue. Molecular weights are indicated in kDa. H (Heavy Chain), L (Light chain). B) Titration ELISA of purified antibodies. 0.1 μg/ well of RpoB or TopoI were adsorbed to microtiter plates and serial dilutions (1/3) of sera were tested. (TIF) [file pgen.1009542.s001.tif]

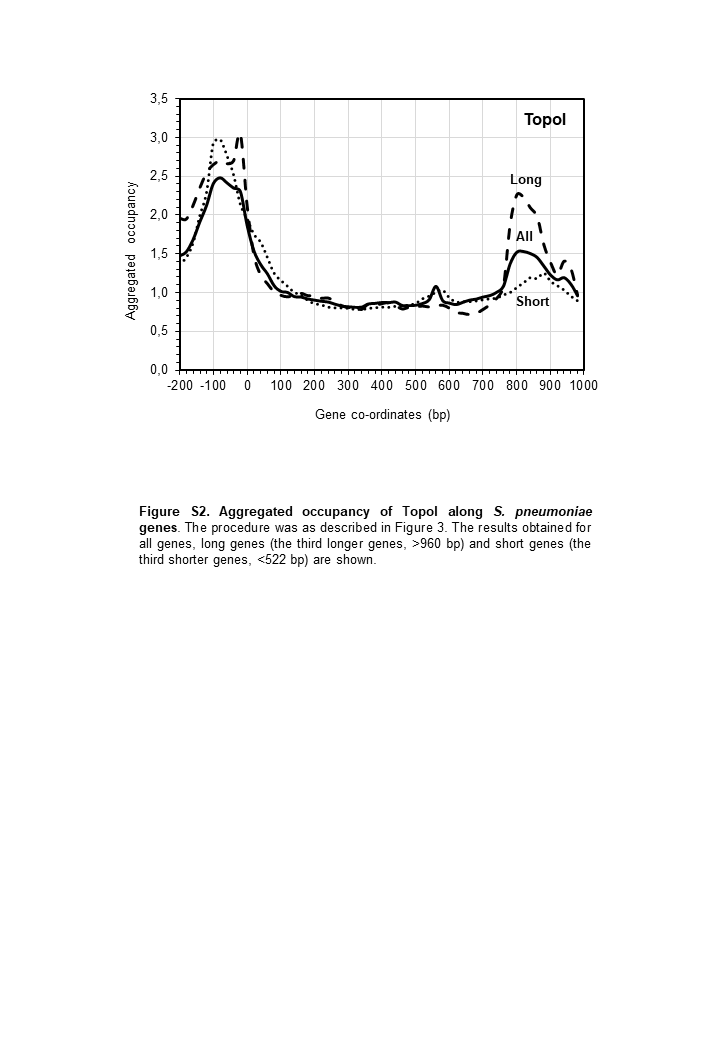

Supplement: S2 Fig — The procedure was as described in Fig 3. The results obtained for all genes, long genes (the third longer genes, >960 bp) and short genes (the third shorter genes, <522 bp) are shown. (TIF) [file pgen.1009542.s002.tif]
