# Supplementary material for: Genome-wide proximity between RNA polymerase and DNA topoisomerase I supports transcription in Streptococcus pneumoniae
Source: PLoS Genet. 2021 Apr 30;17(4):e1009542. doi: 10.1371/journal.pgen.1009542 (PMC8115823; doi:10.1371/journal.pgen.1009542)
Supplement: Attachment — Submitted filename: Response to reviewers V5.docx [file pgen.1009542.s004.docx]

Dear Editor,

Thank you very much for kindly considering our study for publication in PLOS Genetics. Please, find attached a copy of the revised manuscript. We appreciate the suggestions of the reviewers, which have greatly contributed to improve the quality of our article. The manuscript has been modified in accordance. We have addressed the reviewer’s concerns in a point-by-point manner.

**Reviewer #1:**

1. *The basis of change in TopoI promoter occupancy sites in SCN treated cells remains unclear. SCN is a phenanthroline alkaloid that is expected to act as an DNA intercalator. DNA intercalation may affect DNA supercoiling and TopoI binding to chromosomal DNA loci. To further understand effect of SCN treatment on TopoI occupancy of different promoters, there should be a control experiment with a similar DNA intercalator that does not act as a strong TopoI inhibitor.*

We have performed a ChiP-Seq experiment in cultures treated with ethidium bromide (EtBr), a typical DNA intercalant compound. To compare with SCN-treated cultures, which were treated at 0.5× MIC, we have also used a concentration equivalent to 0.5× MIC (0.25 µg/ml, 0.65 µM) of ethidium bromide. EtBr is a good candidate for this experiment, since we have previously shown that it does not inhibit the pneumococcal TopoI enzyme (reference 5 of the manuscript). The occupancy of gene promoters by RNAP and TopoI was indeed affected by EtBr (Figure 1). In the presence of EtBr, 763 promoters were occupied by RNAP. This occupancy was higher than in untreated cultures or cultures treated with SCN (435 and 432, respectively). Similarly, the number of gene promoters occupied by TopoI was higher in EtBr-treated cultures (462) than in untreated (356) or SCN-treated cultures (335). These results indicate that DNA intercalation of EtBr affects RNAP and TopoI occupancy. However, SCN occupancy showed a different pattern than that of EtBr occupancy (Figure 1). Common promoters occupied by RNAP both under EtBr and under SCN treatments were 323, which represent 42.3% and of the total promoters occupied in the presence of EtBr and 74.7% in the case of SCN. With respect TopoI, promoters occupied both under EtBr and under SCN treatments were 251, representing 54.3% and 74.9% of those occupied in the presence of EtBr and SCN, respectively. In addition, the analysis of the aggregated occupancy along genes of both RNAP and TopoI in the presence of EtBr (Figure 2) showed that this compound affected the pattern compared to untreated cultures. In addition, the pattern of occupancy in SCN-treated cultures was radically different to that of EtBr-treated cultures. These results indicate that the influence of SCN on the positioning of RNAP and TopoI observed in our work is not the consequence of a putative intercalating effect.

2. *As the distance between ATG and transcription termination site increases with longer lengths of ORF, requirement of relieve of transcription driven Sc during elongation should take TopoI further away from the ATG. This should be analyzed to confirm that TopoI has a significant role in S. pneumoniae transcription elongation.*

We have analysed TopoI occupancy along long and short genes and included the results in the new supplementary Figure S2. Results are described on page 8, second paragraph as follows: “As RNAP, TopoI was also preferentially positioned upstream the ATG codon in untreated cultures (Fig 3B). However, its occupancy along the gene body was lower than that of RNAP and a clear effect of the level of gene expression was no observed. As TopoI relieves negative Sc generated during transcription, it could be expected that as the distance between ATG and transcription termination site increases in long genes, a higher presence of TopoI at positions further away from the ATG would be detected. We observed that TopoI occupancy was high around the end of long genes (the third longer genes, >960 bp) compared to all genes or short genes (the third shorter genes, <522 bp) (Fig S2).”.

Discussion of these results is included on page 12, end of second paragraph: “We observed that TopoI is more enriched at gene promoters and at the end of long genes than in the gene body (Fig 3B and S2). This might be related to the higher presence of R-loops at these positions, as observed in eukaryotes [43]”.

3. *It is not known if the -10-extended promoter sequence motif is recognized specifically by TopoI, or if this sequence binds TopoI at high frequency because it is found upstream of highly transcribed divergent genes. The in vitro binding affinity of TopoI to this -10-extended sequence should be investigated.*

A DNA fragment corresponding to the promoter region of *codY* gene (P*_codY_*) (from -81 to +75 relative to ATG) was used to check binding of Topo I and RNAP. This fragment contained TopoI motif 2 and the -35 and -10 extended promoter sequences. This sequence was chosen among those showing a TopoI binding peak with high peak score in ChIP assays. This DNA was obtained by PCR using one of the oligonucleotides biotinylated at the 5’ end (5’-Biotin-GTCAATAAGGAATTTTCAGA-3’). After trying unsuccessfully different Biacore settings, that included protein captured by Ni+ chips or immobilized His-Tag antibody or direct protein immobilization by amine protocol, the 166-bp DNA-biotin was finally captured as a ligand (31 response units, RUs) *via* streptavidin attached to the surface of a CM5 sensor chip (1913 RUs at the reference surface and 1868 RUs at the active surface). RNAP and TopoI analytes were dialyzed against the running buffer (20 mM Tris-HCl, pH 8, 100 mM KCl, 10 mM Mg_2_Cl, 1 mM DTT) and binding of RNAP and TopoI at different concentrations, from 12.5 nM to 2000 mM, was tested. Kinetic and affinity measurements were determined using reference-subtracted data. In the case of TopoI, binding was observed only at low concentration of the protein (125 nM). Higher concentrations showed non-specific binding to the reference surface, making difficult to calculate robust affinity determinations. Several approaches to avoid this non-specific binding were tested. These included: higher salt concentration (200 mM KCl); addition of BSA as a surfactant (0.01, 0.05 and 1%); and attachment of TopoI to the sensor surface, via a His-Tag antibody or Ni^2+^. Addition of BSA or higher salt concentration did not prevent non-specific binding. Binding to His-tag antibody or to Ni^2+^ resulted in a weak attachment. Direct binding of TopoI to CM5 sensor surface was also assayed, however DNA was unable to bind to the immobilized TopoI. In the case of RNAP, a range of concentrations from 12.5 nM to 800 nM were tested, showing binding kinetics that allowed to calculate a dissociation constant (K_D_) of 98 nM. Graphs of TopoI and RNAP binding to P*_codY_* at comparable concentrations (125 and 100 nM, respectively) are shown.

P*_codY_*/ TopoI

56.2 RUs

125 nMTopoI

Buffer

P*_codY_*/ RNAP

79.0 RUs

100 nM RNAP

Buffer

0

100

200

300

0

50

100

150

Time (s)

R

e

s

p

o

n

s

e

(

R

U

s

)

0

100

200

300

0

50

100

150

Time (s)

R

e

s

p

o

n

s

e

(

R

U

s

)

**Figure 3. TopoI and RNAP bind to P*_codY_*.** Sensorgrams for SPR measurements.

These SPR results indicate that both RNAP and TopoI bind to the promoter sequence of P*_codY_* and they interact with the DNA.

We have performed EMSAs (Electrophoretic Mobility-Shift Assays) using the same P*_codY_* fragment described above. Binding reactions were carried out in 20 µl containing 2 ng of the biotin-labelled fragment. The reaction buffer was the same used in SPR experiments and 0.2 µg BSA and 1µg poli dI:dC. Proteins were added at the indicated concentrations (Figure 4). Samples were incubated 20 min at 25ºC and resolved in 5% polyacrylamide gels. Two RNAP-P*_codY_* complexes were observed, while no retarded complexes were detected with TopoI, even at high concentrations.

**Figure 4. TopoI and RNAP bind to P*_codY_*.**

The resolution of these apparent discrepancies between the SPR and EMSA results concerning TopoI would require a significant number of additional experiments. Our ChIP-seq results, however, clearly show that TopoI is located frequently at the promoter region of several genes where the -10 Ext sequence is located. In the manuscript we do not conclude that TopoI specifically recognizes this sequence, since it cannot be ruled out, as referee #1 points out, TopoI is preferentially positioned at the promoters of highly expressed genes where the -10 Ext sequence is often located.

We believe that *in vivo* binding of TopoI to gene promoters likely depends not only on DNA sequence but also on DNA supercoiling. *In vitro* TopoI binding to linear DNA would not reflect the *in vivo* binding.

4. *The authors noted that residues in E. coli TopoI proposed for interaction with RNAP are not conserved in S. pneumoniae. It has been reported that M. tuberculosis TopoI utilized residues different from E. coli Topo I to interact with RNAP.* [*https://pubmed.ncbi.nlm.nih.gov/28843989/*](https://pubmed.ncbi.nlm.nih.gov/28843989/)*. This evidence that different bacterial species may utilize distinct mechanism for direct interaction between TopoI and RNAP should be included in the Discussion.*

Thank you for this suggestion, we have changed this paragraph and included the indicated reference (page 11, end of last paragraph): “Likewise, scanning the occupancy through genes showed a close proximity between TopoI and RNAP (Fig 3A and 3B). This proximity could be mediated by a physical interaction between TopoI and RNAP, which is supported by the observed tight in vitro interaction between both proteins (Fig 1B). RNAP-TopoI in vitro physical interaction has also been reported for its homologs in *E. coli* [30, 31]. We observed a tighter association in *S. pneumoniae* (KD=27 nM) than that observed in *E. coli* (KD=93 nM), and a slower dissociation, probably because we used the RNAP holoenzyme, while only the core enzyme was used in *E. coli*. In addition, structure-based modelling of RNAP-TopoI interaction in *E. coli* has identified the residues of TopoI and subunit RNAP β' [31] involved in the interaction.”

5. *On page 12 of Discussion, the authors proposed that “TopoI plays a role in head-on genes, likely resolving torsional stress generated when transcription and replication collide”. This proposed role is not clear and needs to be further explained, as positive Sc is generated ahead of both transcription and replication, and TopoI cannot relax positively supercoiled DNA.*

This has been changed to: “This indicates that TopoI plays a role in head-on genes, likely favouring their transcription that is compromised due to the accumulation of positive Sc when transcriptional and replication forks approach in opposite directions”.

*Other minor comments:*

Thank you for the careful inspection of the manuscript. All suggested corrections have been made.

**Reviewer #2:**

*Introduction*

1. *“In S. pneumoniae, the transcriptomic response to relaxation by inhibition of gyrase with novobiocin or by inhibition of TopoI by SCN, has revealed Sc-domains.” – Surely this is a mis-phrasing as inhibition of TopoI should prevent relaxation.*

This has been changed to: “In S. pneumoniae, the transcriptomic response to relaxation by inhibition of gyrase with novobiocin or to hypernegative Sc by inhibition of TopoI with SCN, has revealed Sc-domains”.

2. “*The increase in Sc mediated by SCN caused modulates the transcription of 10% of the genome*”

The word “caused” has been removed from the sentence.

3. *“AT-content correlates with UP domains.” Is this AT high or AT low content?*

The sentence has been changed to “AT-content is higher in UP domains than in DOWN domains”.

*Major Comments*

*Results*

*P7 “The effect of SCN on the promoter occupancy by RNAP was practically negligible (compare Fig 2C and 2F), since most of the RNAP-enriched promoters remained enriched under SCN treatment (Fig 2F).”(I presume this is actually “2E and 2F”?) Whilst the number of RNAP occupied promoters remained similar after SCN treatment 25% of the genes changed – this is hardly negligible. Did it affect the proportions of the enriched groups?*

This has been changed to: “The effect of SCN on the promoter occupancy by RNAP was lower than that of Rif (compare Fig 2E and 2F). Moreover, SCN treatment did not change the proportional representation of gene functional classes.”

*It is unclear as to whether the specificity of the TopoI binding motifs has been determined. We are told that Motif 2 is a -10 extended sequence and is “commonly found at pneumococcal promoters “ – as Motif 2 is only found at 18% of TopoI peaks how common is this Motif at promoter sequences where TopoI does not show enhanced binding?*

We have carried out the suggested analysis. Findings has been included on page 10, third paragraph: “We analyzed how common motif 2 was at promoters not enriched by TopoI (ER<2) compared to TopoI-enriched promoters (ER>2). We used FIMO program to search for significant occurrences (P-value<1E-6) of this motif in each subset of promoters. Motif 2 was significantly more frequent (P-value <0.0001) at TopoI-enriched promoters (15.7%, 56/356) than at the promoters not enriched by TopoI (5.1%, 87/1687)”.

*Discussion*

*“Our ChiP experiments showed that RNAP preferentially located at promoter regions (Fig 2A) of highly expressed genes, and that promoters bound by both TopoI and RNAP had higher levels of expression than those bound by only RNAP (Fig 2A). These results indicate that TopoI plays a role in the activation of transcription.”
It is not immediately obvious to me why this supports TopoI being directly involved in the activation of transcription rather than it just being recruited more often at these sites where it is required more because transcription is higher.*

We have changed the sentence to: “These results might indicate that TopoI plays a role in the activation of transcription”.

*Also, in work in Mycobacteria the TopoI was shown by ChiP to bind relatively evenly across the whole genome (Rani et al NAR 2019) but that topoI only showed peaks of cleavage activity at the highly expressed promoters – which is different to the finding of Ahmed also in mycobacteria (Ahmed PLoS Genetics 2017) shows simila binding of topoI and gyrase. Is there any suggestion for why this should be different in the Pneumococcus?*

We consider that our results of TopoI occupancy do not differ much from those by Rani and Nagaraja in Mycobacteria. In both cases, TopoI preferentially locates at the promoter region of highly expressed genes and declines downstream this position (our Fig 3B and Fig 4A of Rani and Nagaraja). Interestingly, TopoI cleavage activity is higher downstream the TSS in Mycobacteria and low at the promoter region. Further experiments on the genome-wide cleavage activity of TopoI in the Pneumococcus are needed to ascertain differences and similarities of the mechanism of action of TopoI in both bacteria.

*“In addition, structure-based modelling of RNAP-TopoI interaction in E. coli has identified the residues of TopoI and subunit RNAP B' [31] involved in the interaction. However, none these residues are conserved in the pneumococcal proteins. More structural studies in pneumococcus are needed to characterize this interaction.”*

*Whilst I appreciate that the residues identified in the E. coli RNAP-TopoI interaction are not present in Pneumococcus it is surprising that you have not commented on the fact that Rifampicin, which in your data (Fig 1) inhibits the TopoI-RNAP interaction, does so by binding the RNAP β subunit (which is also the site of the interaction shown in E. coli). Could this simply be the mechanism of Rifampicin interference with RNAP-TopoI binding?*

Thank you for this suggestion. It has been included in the following paragraph: “Likewise, it has been reported that the residues utilized by *M. tuberculosis* TopoI to interact with RNAP are different from those used by *E. coli* TopoI [31, 39]. This shows that different bacterial species utilize distinct mechanisms for TopoI-RNAP interaction. Rif, which inhibits the pneumococcal RNAP β subunit [40], prevented TopoI-RNAP interaction (Fig 1C), suggesting a role for RNAP β subunit in such interaction. More structural studies in the pneumococcus are needed to characterize this interaction.”

*In the discussion of the DNA binding motifs for the pneumococcal topoI the paper would significantly benefit from discussing the papers defining the target motifs for pneumococcal topoIV and gyrase (Leo et al 2005 JBC, Laponogov et al., 2009 NatStucBiol and 2010 PLoS One). Also, Sutormin NAR 2019 could be a good contribution to the discussion of the DNA motifs, even if this is E. coli, but the concept of a larger (130 bp) target motifs is interesting to include in the discussion.*

We agree that the studies on the molecular interactions between DNA topoisomerases and DNA are relevant. Although the knowledge of the interactions of the pneumococcal type II topoisomerases (TopoIV and gyrase) is of basic and clinical relevance, we do not consider it essential for the present study.

*The link to data in NCBI does not seem to find the data. (Though this could be NCBI!)*

Data are now available at https://www.ncbi.nlm.nih.gov/bioproject/PRJNA656439

*Minor comments and typos*

*The manuscript would be improved immensely by some additional editing work. The abstract and summary in particular appear to have been cut to fit the word limit without enough checking for continued clarity.*

*There are quite a lot of typos – this is not an exclusive list.*

Thank you for your careful corrections, we have made all changes. In addition, the English has been corrected by P. A. Lazo, from the Institute of Molecular Biology and Cellular of Cancer. CSIC-University of Salamanca.

**Reviewer #3:**

1. *Direct role of TopoisomeraseI in the activation of transcription is claimed based on a single experiment on RNAP interaction with the topoisomerase and co-localization of both the enzymes at the promoter region. Interaction should have been studied at least with yet another technique. If SPR assays were conducted with immobilized DNA and then passing the two proteins sequentially would have been a more insightful experiment to see their direct interaction on DNA. In this experiment, one can perform order of addition experiment to understand the interaction more meaningfully. Which sub -unit of RNAP is interacting with TopoisomeraseI ? A large number of studies have been published on RNAP sub unit interaction with various proteins using a whole range of techniques including SPR. Also, the experiment described does not provide information on what fraction of TopoisomeraseI and RNAP interact with each other in the cell. Is it a small fraction or large fraction would be important to know. As they have high quality antibodies (see later point), it is easy to estimate the extent of interaction.*

*3. Both Topoisomerase I and RNAP binding to the same site at the promoter is problematic. If indeed both are recruited to the same site, they would compete out each other instead of cooperating for transcription. First, one can test and validate binding of both the enzymes to the extended – 10 containing promoter sequences by EMSA to confirm the consensus site they derived from ChiP Seq. If indeed they are bound, one has to displace the other! RNAP binds a large region at the promoters and it would be interesting to know where Topoisomerase I binds.- upstream , downstream or on RNAP.*

A DNA fragment corresponding to the promoter region of *codY* gene (P*_codY_*) (from -81 to +75 relative to ATG) was used to check binding of Topo I and RNAP. This fragment contained TopoI motif 2 and the -35 and -10 extended promoter sequences. This sequence was chosen among those showing a TopoI binding peak with high peak score in ChIP assays. This DNA was obtained by PCR using one of the oligonucleotides biotinylated at the 5’ end (5’-Biotin-GTCAATAAGGAATTTTCAGA-3’). After trying unsuccessfully different Biacore settings, that included protein captured by Ni+ chips or immobilized His-Tag antibody or direct protein immobilization by amine protocol, the 166-bp DNA-biotin was finally captured as a ligand (31 response units, RUs) *via* streptavidin attached to the surface of a CM5 sensor chip (1913 RUs at the reference surface and 1868 RUs at the active surface). RNAP and TopoI analytes were dialyzed against the running buffer (20 mM Tris-HCl, pH 8, 100 mM KCl, 10 mM Mg_2_Cl, 1 mM DTT) and binding of RNAP and TopoI at different concentrations, from 12.5 nM to 2000 mM, was tested. Kinetic and affinity measurements were determined using reference-subtracted data. In the case of TopoI, binding was observed only at low concentration of the protein (125 nM). Higher concentrations showed non-specific binding to the reference surface, making difficult to calculate robust affinity determinations. Several approaches to avoid this non-specific binding were tested. These included: higher salt concentration (200 mM KCl); addition of BSA as a surfactant (0.01, 0.05 and 1%); and attachment of TopoI to the sensor surface, via a His-Tag antibody or Ni^2+^. Addition of BSA or higher salt concentration did not prevent non-specific binding. Binding to His-tag antibody or to Ni^2+^ resulted in a weak attachment. Direct binding of TopoI to CM5 sensor surface was also assayed, however DNA was unable to bind to the immobilized TopoI. In the case of RNAP, a range of concentrations from 12.5 nM to 800 nM were tested, showing binding kinetics that allowed to calculate a dissociation constant (K_D_) of 98 nM. Graphs of TopoI and RNAP binding to P*_codY_* at comparable concentrations (125 and 100 nM, respectively) are shown.

P*_codY_*/ TopoI

56.2 RUs

125 nMTopoI

Buffer

P*_codY_*/ RNAP

79.0 RUs

100 nM RNAP

Buffer

0

100

200

300

0

50

100

150

Time (s)

R

e

s

p

o

n

s

e

(

R

U

s

)

0

100

200

300

0

50

100

150

Time (s)

R

e

s

p

o

n

s

e

(

R

U

s

)

**Figure 3. TopoI and RNAP bind to P*_codY_*.** Sensorgrams for SPR measurements.

These SPR results indicate that both RNAP and TopoI bind to the promoter sequence of P*_codY_* and they interact with the DNA.

We have performed EMSAs (Electrophoretic Mobility-Shift Assays) using the same P*_codY_* fragment described above. Binding reactions were carried out in 20 µl containing 2 ng of the biotin-labelled fragment. The reaction buffer was the same used in SPR experiments and 0.2 µg BSA and 1µg poli dI:dC. Proteins were added at the indicated concentrations (Figure 4). Samples were incubated 20 min at 25ºC and resolved in 5% polyacrylamide gels. Two RNAP-P*_codY_* complexes were observed, while no retarded complexes were detected with TopoI, even at high concentrations.

**Figure 4. TopoI and RNAP bind to P*_codY_*.**

The resolution of these apparent discrepancies between the SPR and EMSA results concerning TopoI would require a significant number of additional experiments. Our ChIP-seq results, however, clearly show that TopoI is located frequently at the promoter region of several genes where the -10 Ext sequence is located. In the manuscript we do not conclude that TopoI specifically recognizes this sequence, since it cannot be ruled out, as referee #1 points out, TopoI is preferentially positioned at the promoters of highly expressed genes where the -10 Ext sequence is often located.

We believe that *in vivo* binding of TopoI to gene promoters likely depends not only on DNA sequence but also on DNA supercoiling. *In vitro* TopoI binding to linear DNA would not reflect the *in vivo* binding.

In relation to the RNAP subunits interacting with TopoI, as suggested by other reviewers, we have modified this part of the discussion, by adding the following text: “Likewise, it has been reported that the residues utilized by *M. tuberculosis* TopoI to interact with RNAP are different from those used by *E. coli* TopoI [31, 39]. This shows that different bacterial species utilize distinct mechanisms for TopoI-RNAP interaction. Rif, which inhibits the pneumococcal RNAP β subunit [40], prevented TopoI-RNAP interaction (Fig 1C), suggesting a role for RNAP β subunit in such interaction. More structural studies in the pneumococcus are needed to characterize this interaction.”

2. *Topoisomerase I binding to the promoter does not suggest that it activates RNAP. To test the activation model, increase in complex formation or stability of the complex as they claim, the authors need to check the influence of Topoisomerase I on RNAP closed complex, open complex formation and if necessary(if the enzyme has no effect on the earlier steps) promoter clearance and abortive initiation assays. To test the effect of the enzyme on RNAP –promoter complex stability, complex decay experiments need to be performed. Without these assays, the activation model is a conjecture.*

We have found that TopoI and RNAP physically interact in vitro and that both enzymes preferentially co-localize in the promoters of highly expressed genes in vivo. In addition, the TopoI peaks are located close to the ATG codon and contain the extended -10 motif present in multiple promoters in *S. pneumoniae*. These results, together with the relative positioning of the observed TopoI and RNAP peaks and the study of the use of inhibitors of TopoI or RNAP, although they do not demonstrate, allow us to propose a role played by TopoI in transcription.

4. *Figures- Fig1A- Purified Topoisomerase I has at least 10 additional proteins. If silver stained, more bands will be seen. Similarly, RNAP preparation has 5 additional bands, some of which are of lower intensity. This is problematic as in the described SPR amino coupling is done and some of these proteins may interfere resulting in erroneous interpretation of the data. Fig1B is very poor representation of RNAP. With the availability of crystal structure of RNAP from various species of bacteria, one can draw a better, a more representative cartoon of the enzyme. As such, I do not see the need for the figure as it is not serving any purpose. If TopoisomeraseI interaction was shown with a subunit of RNAP, perhaps, the cartoon would serve some purpose.*

Although purified Topo I shows some contaminants, they do not affect the activity of the protein (reference 5). RNAP has been purified following the method of Lee and Morrison (reference 49), which shows a good transcription activity *in vitro*. The inhibition of the RNAP-TopoI complex formation by both rifampicin and SCN (Figure 1C), specific inhibitors of these enzymes, supports the binding of RNAP and TopoI in the complex.

Fig1B has been removed.

*Supplementary figure: Antibody purification is described in detail. There is no ‘High’ chain in an antibody. Replace with ‘heavy’chain.*

‘High’ has been replaced by ‘Heavy’. Thank you for the correction.

5. *ChiP Seq experiments: Some of the data are clear whereas others beg some explanation. Some others are difficult to explain. For example, why treatment with Topoisomerase I inhibitor SCN should alter the RNAP occupancy? If TopoI activity is necessary for transcription activation, the inhibitor (SCN) treatment should alter RNA transcripts levels of specific set of genes.*

We understand that reviewer 3 believes SCN should only alter the amount of RNA transcripts, since TopoI would be required for RNAP activation, but that it should not change the pattern of occupancy of RNAP along the genes (Fig 3E and 3G). We observed that when TopoI is inhibited with SCN, the occupancy of RNAP upstream of the ATG codon declines. Our interpretation is that TopoI would be required for the correct stability of RNAP at the promoter. We also observed that RNAP accumulates more in the transcribed region in the presence of SCN. Our interpretation is that TopoI would also contribute to the correct displacement of RNAP, so that transcription complexes that are moving through the gene body have difficulty progressing in the presence of SCN. We suggest that this is probably due to the accumulation of negative Sc when TopoI is inhibited, which is known to generate more R-looping that makes elongation difficult.

*Further, I am not able to understand Rifampicin affecting RNAP occupancy as its action is after the formation of the ternary complex and early step of elongation(authors have written and cited this information).*

As mentioned in the manuscript, it has been shown that the predominant effect of Rif at high concentration is “to directly block the path of the elongating RNA transcript at the 5’ end when the transcript becomes either 2 or 3 nt in length” (Campbell et al., 2001). The experiments performed in Campbell’s study used high Rif concentrations, in the order of 6.8-fold the MIC. However, we used much lower Rif concentrations, so that some elongation was likely allowed at the subinhibitory concentrations of 0.5× MIC. Consistently, using higher Rif concentration (1x MIC), RNAP was mainly trapped at the ATG codon (Fig. 3C and 3G). As we mention in the manuscript, a similar effect has been also observed previously in *E. coli* in ChIP-chip experiments using antibodies against RpoB or the sigma subunit (Davis et al, 2011).

*Additional points/corrections:*

*Fig 1D – Why SCN or Rifampicin addition should change RUs?
Many sentence and typographical errors-*

*Author summary – sentence 2 and others.*

*Introduction: ...caused...modulates...; Relaxing topoisomerase?*

*....generated respectively, behind and ahead of the moving RNAP*

*...which, otherwise, would difficult transcription elongation*

*Results: SCN dropped the vale( ??) to 40.9 units*

*Many others including a sub title in the Results section – ‘ Identification of TopoI and RNAP peaka and identification of binding motifs’*

Thank you for your careful corrections, we have made all changes, in addition, the English has been corrected by P. A. Lazo, from the Institute of Molecular Biology and Cellular of Cancer. CSIC-University of Salamanca

**Have all data underlying the figures and results presented in the manuscript been provided?**
Large-scale datasets should be made available via a public repository as described in the *PLOS Genetics* [data availability policy](http://journals.plos.org/plosgenetics/s/data-availability), and numerical data that underlies graphs or summary statistics should be provided in spreadsheet form as supporting information.

*Reviewer #1:* ***No:*** *No data is available through the links provided* [*https://dataview.ncbi.nlm.nih.gov/object/PRJNA656439?reviewer=hrj007r86ulcrnktia18*](https://dataview.ncbi.nlm.nih.gov/object/PRJNA656439?reviewer=hrj007r86ulcrnktia18)

Data are now available at https://www.ncbi.nlm.nih.gov/bioproject/PRJNA656439

[*http://microbes.ucsc.edu/cgibin/hgTracks?hgS_doOtherUser=submit&hgS_otherUserName=Pablo_Hernandez&hg*](http://microbes.ucsc.edu/cgibin/hgTracks?hgS_doOtherUser=submit&hgS_otherUserName=Pablo_Hernandez&hg) *S_otherUserSessionName=S_pneumoniae_ChIP-seq*

*I assume that this is a technical issue, and data will become available*

We have confirmed that he link to the [UCSC genome browser](https://genome.ucsc.edu/) session displaying the uploaded sequence tracks is available at: http://microbes.ucsc.edu/cgi-bin/hgTracks?hgS_doOtherUser=submit&hgS_otherUserName=Pablo_Hernandez&hgS_otherUserSessionName=S_pneumoniae_ChIP-seq

*Reviewer #2: Yes*

*Reviewer #3: Yes*

We sincerely hope that the revised version is now acceptable for publication in PLOS Genetics.

Sincerely yours,

Adela G. de la Campa

Unidad de Genética Bacteriana
Centro Nacional de Microbiología
Instituto de Salud Carlos III
28220 Majadahonda, Madrid, Spain
